# A Mesenchymal Stromal Cell Gene Signature for Donor Age

**DOI:** 10.1371/journal.pone.0042908

**Published:** 2012-08-23

**Authors:** Hugo Alves, Jetty van Ginkel, Nathalie Groen, Marc Hulsman, Anouk Mentink, Marcel Reinders, Clemens van Blitterswijk, Jan de Boer

**Affiliations:** 1 Department of Tissue Regeneration, MIRA Institute for Biomedical Technology and Technical Medicine, University of Twente, Enschede, The Netherlands; 2 Information and Communication Theory Group, Faculty of Electrical Engineering, Mathematics and Computer Science, Delft University of Technology, Delft, The Netherlands; RWTH Aachen University Medical School, Germany

## Abstract

Human aging is associated with loss of function and regenerative capacity. Human bone marrow derived mesenchymal stromal cells (hMSCs) are involved in tissue regeneration, evidenced by their capacity to differentiate into several lineages and therefore are considered the golden standard for cell-based regeneration therapy. Tissue maintenance and regeneration is dependent on stem cells and declines with age and aging is thought to influence therapeutic efficacy, therefore, more insight in the process of aging of hMSCs is of high interest. We, therefore, hypothesized that hMSCs might reflect signs of aging. In order to find markers for donor age, early passage hMSCs were isolated from bone marrow of 61 donors, with ages varying from 17–84, and clinical parameters, *in vitro* characteristics and microarray analysis were assessed. Although clinical parameters and *in vitro* performance did not yield reliable markers for aging since large donor variations were present, genome-wide microarray analysis resulted in a considerable list of genes correlating with human age. By comparing the transcriptional profile of aging in human with the one from rat, we discovered follistatin as a common marker for aging in both species. The gene signature presented here could be a useful tool for drug testing to rejuvenate hMSCs or for the selection of more potent, hMSCs for cell-based therapy.

## Introduction

The average human life span has been increasing over the last decades, mainly due to the continuous advances in medical research but also due to the improvements of general life conditions. Unfortunately, human aging is associated with disease, loss of regenerative capacity and loss of function and, therefore, there has been an increasing crisis in organ transplantation but also in elderly-related diseases like Parkinson and Alzheimer. Also concomitant with an increasing aging of the population, elderly-related bone fractures increased significantly and since there is an evident loss of bone regenerative capacity with age, the amount of patients which will potentially benefit from stem cell based therapies keeps on increasing, which strengthens the need for further investigation on age-related differences in stem cell capacity. Focus has therefore, been placed in cell-based regenerative therapies, with stem cells as potential sources, with the hope to repair, restore or reduce these pathologies. Among stem cells, human mesenchymal stromal cells have been considered as the gold standard for cellular therapy. Not only because they are multipotent [1], easy to isolate and can be expanded *in vitro*, but also because they secrete trophic and immunomodulatory factors, giving them therapeutic qualities and making them more appropriate for allogeneic transplantation [2]. Furthermore, hMSCs have established their value in clinical trials [3], [4] and several others are currently being undertaken. Tissue maintenance and regeneration is dependent on stem cells and therefore, any loss in number or functionality due to aging will likely have a profound effect on our regenerative capacity [5].

Recently, Zhuo et al. found an equal contribution of donor age and recipient age to the efficacy of rat MSC-based therapy, with an overall decline in efficacy with age of the donor and the recipient, suggesting stem cells are indeed influenced by the process of aging [6], [7]. For muscle, on the other hand, the age of the microenvironment and niche, in which progenitor cells reside, has a far greater influence than the intrinsic proliferative potential of the progenitor cells. Loss of regeneration in muscle is, at least partly, due to loss of Notch signaling, which could be restored by exposure of progenitor cells to a young microenvironment [8]. For MSCs the niche they reside in, the bone marrow, is less well-defined and little is known about the effect of aging on this microenvironment. The bone marrow is a complex three-dimensional structure comprising of hematopoietic cells, MSCs, adipocytes, endothelial cells and pericytes, all of which are influenced by aging [9]. Since MSCs interact with several cell types including hematopoietic cells (HSCs), which were reported to be required to stimulate MSCs colony formation [10], it is important to consider that age-related changes in HSCs [11] might impact MSC function as well.

An important aspect is then to determine whether MSCs are aging themselves (intrinsic aging) or whether it is the environment that is causing them to senesce by not providing the right signals (extrinsic aging). Since MSCs reside in a very complex environment and since most of the bone marrow niche components (matrix, HSCs, and other cells) show age-related changes [9], all these factors can easily interfere with the biological properties of MSCs.

Understanding the molecular pathways involved in aging is crucial and will help in the development of cell-based regeneration strategies.

Since different species age at different rates and possess distinct maximum lifespan, at least part of the process of aging is suggested to be controlled by gene expression. Human lifespan, determined by genetics and external factors such as injuries and lifestyle, is thought to be inheritable for up to 25% [12]. Lifespan in centenarians has an even larger genetic component [13], [14]. With microarray techniques improving in specificity and accuracy, extensive gene studies become of high interest in the search for markers for aging and the understanding of the genetics behind the process. The accumulation of DNA damages has been introduced as a cause of loss of multipotency of hMSCs *in vitro* [15]. Moreover, we and others reported on defects in DNA repair pathways which have been associated with premature aging and reduced longevity in mice with a genetic mutation similar to the human disorder trichothiodystrophy (TTD) [16]. However, the link between *in vitro* senescence and *in vivo* aging remains unclear.

While some authors have already correlated various tissue-specific gene expression profiles with aging, such as: human brain [17], human muscle [18] or human kidney [19] and some aging patterns are similar between human tissues, much of it is tissue specific [18]. Therefore, in this study we searched for general markers of aging. Here, we present a molecular signature of human aging by performing a genome-wide gene expression analysis in human MSCs as a function of the age of the donors. Moreover, we compared the performance of these aging markers in both aging human and rat MSCs. We furthermore evaluated if these markers obtained also reflected the process of *in vitro* aging.

## Results

### Correlation of biological characteristics and donor age

**Figure 1:**
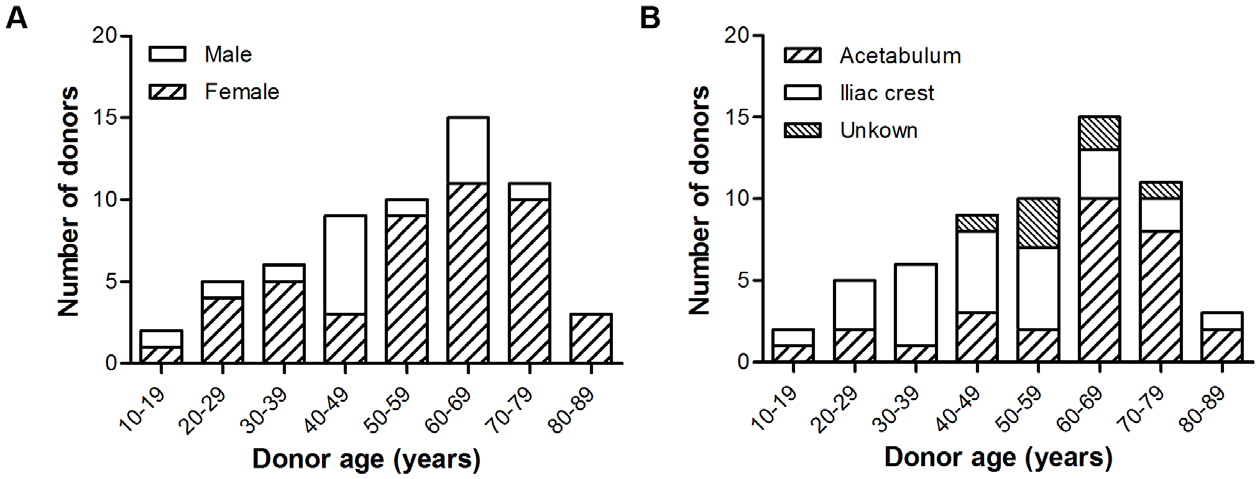
Distribution of the donor population. A) The distribution of sex with age and B) the distribution of site of aspiration with age.

In order to identify molecular markers of the aging process which could provide insights into the molecular mechanism that ultimately limits human lifespan, we have isolated hMSCs from bone marrow of 61 healthy donors to 84 years, with an average of 55 years. The distribution of age, sex and locations of aspiration can be found in Figure 1. The aspirates were put in culture and the cells were identified according to the set of standards proposed by the Mesenchymal and Tissue Stem Cell Committee of the ISCT [20]. Cells were adherent and over 94% expressed CD73 and CD90, 60% expressed CD105, and less than 2% were CD45, CD34, CD11b, CD19 and HLA-DR positive, as determined by flow cytometry in cells from 3 different donors (data not shown).

**Figure 2:**
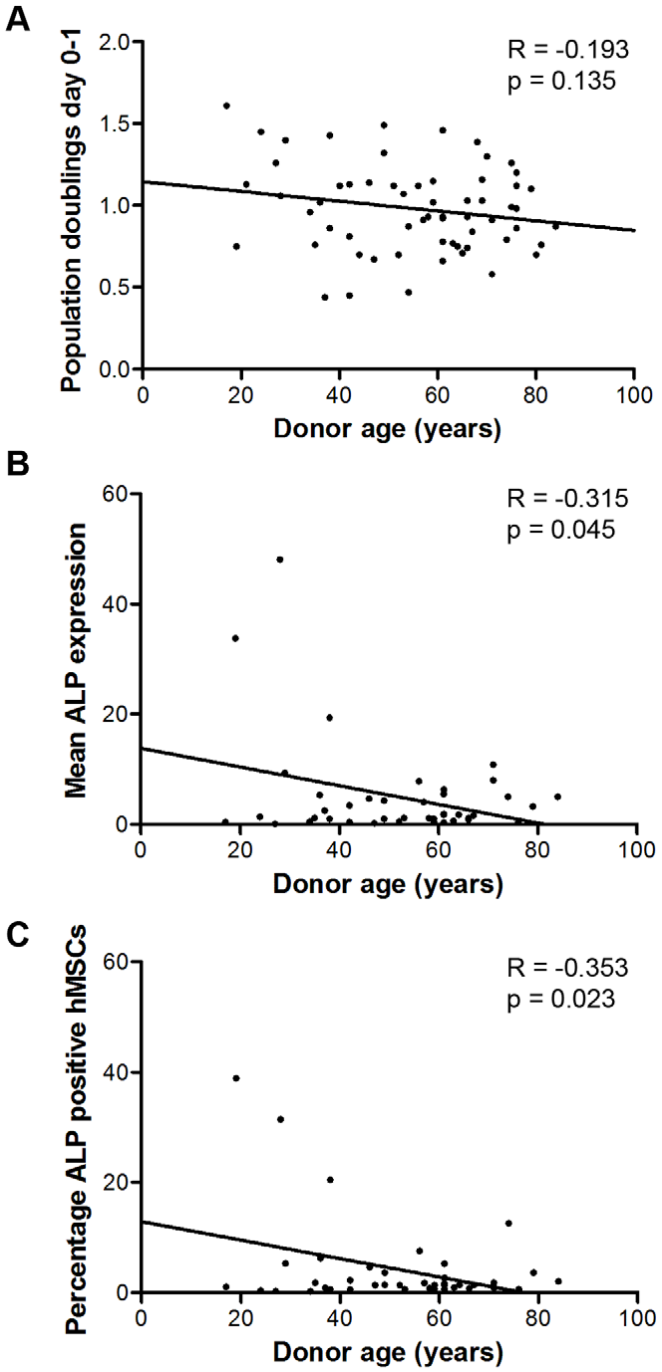
Correlation between biological parameters and donor age. A correlation between age and clinical and biological labels could not be determined for labels such as A) population doublings per day between day 0–1 and bone formation. Correlation with donor age could solely be determined for B) the mean ALP expression and C) the percentage ALP positive cells in basic medium.

We started by examining whether several biological characteristics, such as population doublings, total yield and differentiation capacity of the hMSCs correlated to donor age. The relation between these parameters and donor age were evaluated with Pearson correlations (Table 1; the correlation value is given by the value of R). The data indicates that no strong correlations were identified among the biological characteristics and donor age. Most parameters, such as the number of population doublings between day 0 and day 1 (Figure 2A), did not correlate with donor age. While only the expression of the early osteogenic marker, Alkaline Phosphatase (ALP), did show a slight negative correlation (P<0.05) with donor age, for both the mean expression (Figure 2B) and the percentage of positive cells (Figure 2C), this was mainly due to high expression of ALP in a number of young donors. However, when the osteogenic differentiation capacity was assessed by determining the potency of dexamethasone to induce ALP expression, no correlation was found.

**Table 1:** Pearson correlation of biological characteristics with donor age.

Characteristic	R	Sig.
Yield (mL)	0.167	0.198
Number of nucleated cells per ml	−0,178	0,170
Population doublings, day 0–1	−0.193	0.135
Population doublings, day 1–2	−0.621	0.074
Bone formation	0.033	0.799
Percentage of bone compared to scaffold	0.105	0.420
Percentage of bone contact	0.097	0.458
* Mean ALP expression (control)	−0.315	0.045
Mean ALP expression (dex)	−0.299	0.057
Mean ALP expression (index)	−0.167	0.297
* Percentage ALP positive cells (control)	−0.353	0.023
Percentage ALP positive cells (dex)	−0.217	0.174
Percentage ALP positive cells (index)	−0.143	0.372
Mineralization	−0.129	0.587
Adipogenesis	−0.131	0.570
Cartilage formation (GAG/DNA)	0.423	0.063

* represents p<0.05.

### Genetic markers for donor age

Since gene expression is altered with cellular senescence and since it is currently unknown the extent to which age regulation of gene expression is specific to a particular tissue or common across many, we ought to find whether the gene expression profiles of 61 human bone-marrow-derived hMSCs correlated with the age of their donors. Since stem cells are involved in the regenerative process, which is lost during age, it is likely that hMSCs will also reflect changes during the aging process as well. To perform this genome-wide gene expression analysis, RNA was isolated from undifferentiated hMSCs (passage 2) and hybridized to Human Genome U133A 2.0 Arrays (Affymetrix) comprising of 22,277 probe sets representing 18,400 gene transcripts and variants. During statistical processing, probe sets with sufficient difference in expression (standard deviation across all donors >0.4) were selected. Within the resulting 1653, the correlation between probe sets and donor age was assessed using ANOVA with correction for sex and aspiration site. P values were determined by permutation tests omitting the seven donors from which the aspiration site is “unkown” and false discovery rates were calculated. The probe sets were ranked by significance and the top 70 genes (p<0.01) are listed in Table 2. As an example: gene number 70 in this list (*FADS1*, fatty acid desaturase 1) has a false discovery rate of 0.228 (22.8%) meaning that one can expect a little over 15 genes in this list to be false positive. In addition, this gene has a fold change of −0.012, corresponding to a fold change of −1,2 per 100 years of donor age. We furthermore explored this list of genes by evaluating the enriched gene ontology categories, presented in Table 3. Remarkably, biological process associated GO terms related to neuronal functioning were overrepresented. The “Extracellular region” was the only cellular component related term enriched.

**Table 2:** List of genes correlating in hMSCs with donor age (p<0.01) based on ANOVA.

	Official symbol^d^	p-value^a^	FDR^b^	FC^c^		Official symbol	p-value	FDR	FC
1	SLIT3	0.00002	0.03306	−0.017	36	PDE4DIP	0.00279	0.12288	−0.013
2	FST	0.00009	0.03967	0.019	37	JAG1	0.00279	0.12288	0.016
3	FGFR2	0.00011	0.03967	−0.016	38	COL13A1	0.00290	0.12288	0.016
4	NDNF	0.00012	0.03967	−0.024	39	CXCL12	0.00290	0.12288	−0.012
5	NDNF	0.00012	0.03967	−0.016	40	**SOX4**	0.00301	0.12440	−0.012
6	FGFR2	0.00025	0.06887	−0.013	41	ESM1	0.00334	0.13160	0.018
7	COLEC12	0.00041	0.08430	−0.031	42	IGF2BP3	0.00334	0.13160	0.011
8	**C1R**	0.00042	0.08430	−0.017	43	**SLC1A3**	0.00372	0.13529	−0.015
9	TFPI	0.00050	0.08430	0.014	44	CDH13	0.00372	0.13529	0.013
10	FST	0.00051	0.08430	0.014	45	LIF	0.00372	0.13529	0.017
11	FZD1	0.00059	0.08887	−0.013	46	C21orf7	0.00387	0.13529	0.020
12	OLFML3	0.00087	0.10631	−0.015	47	GLIPR1	0.00387	0.13529	0.012
13	RGS4	0.00097	0.10631	0.034	48	TFPI2	0.00401	0.13529	0.018
14	NRXN3	0.00097	0.10631	0.017	49	**WISP1**	0.00401	0.13529	−0.015
15	CH25H	0.00105	0.10631	−0.029	50	NRN1	0.00451	0.14912	−0.013
16	MATN2	0.00105	0.10631	−0.014	51	GLIPR1	0.00521	0.16564	0.013
17	MGLL	0.00109	0.10631	0.011	52	HMGA2	0.00521	0.16564	0.010
18	BDNF	0.00131	0.11535	0.013	53	MALL	0.00541	0.16564	0.015
19	PTX3	0.00133	0.11535	0.023	54	NR2F2	0.00541	0.16564	0.014
20	ANKRD1	0.00140	0.11562	0.017	55	**COL10A1**	0.00561	0.16864	−0.019
21	TFPI	0.00154	0.11855	0.020	56	**DDIT4**	0.00581	0.17153	−0.020
22	TFPI	0.00174	0.11855	0.020	57	CDH6	0.00601	0.17432	0.017
23	CDH6	0.00180	0.11855	0.026	58	OSR2	0.00621	0.17702	0.012
24	RGS4	0.00187	0.11855	0.034	59	INSIG1	0.00641	0.17962	−0.012
25	MOXD1	0.00193	0.11855	0.016	60	GAS1	0.00701	0.19001	−0.019
26	**MAFB**	0.00193	0.11855	−0.018	61	ANK3	0.00701	0.19001	0.013
27	FOSL2	0.00194	0.11855	−0.015	62	**CXCR7**	0.00751	0.20027	−0.011
28	GPR116	0.00209	0.11855	0.013	63	**DKK1**	0.00776	0.20366	0.022
29	JAG1	0.00209	0.11855	0.017	64	ID2	0.00826	0.20903	−0.011
30	KRT18	0.00218	0.11855	0.033	65	MAFF	0.00835	0.20903	0.011
31	TNFRSF21	0.00237	0.11855	0.020	66	TNFAIP3	0.00835	0.20903	0.010
32	**ZNF365**	0.00237	0.11855	0.011	67	GATA6	0.00901	0.22236	0.019
33	FMO3	0.00243	0.11855	0.028	68	PSG4	0.00935	0.22391	0.011
34	B3GALT2	0.00246	0.11855	0.013	69	**C1S**	0.00935	0.22391	−0.008
35	FMO3	0.00251	0.11855	0.025	70	FADS1	0.00968	0.22858	−0.012

a : p values for genes correlating with age corrected for the confounding effects attributed to the sex of the donor and the aspiration site of the bone marrow. The p values were computed omitting the samples with unknown origin.

b : FDR = the false discovery rate.

c : The slope corresponds to the strength of the correlation. This value can be considered as the fold change per year. (i.e. 0.017 corresponds to a fold change of 1,7 per 100 years).

d : The genes presented in bold are affected by the aspiration site of the bone marrow (acetabulum or iliac crest) (p<0.01).

**Table 3:** GO Enrichment analysis with enrichment for all three GO categories.

Term	Name	FE^a^	P value	Counts^b^
**Biological Process**				
GO:0007411	Axon guidance	10.35	1.12E-04	6
GO:0048667	Cell morphogenesis involved in neuron differentiation	5.31	1.19E-03	7
GO:0065007	Biological regulation	1.32	3.12E-03	42
GO:0000904	Cell morphogenesis involved in differentiation	4.43	3.26E-03	7
GO:0030182	Neuron differentiation	3.35	3.38E-03	9
GO:0048666	Neuron development	3.70	3.79E-03	8
GO:0022008	Neurogenesis	2.68	4.80E-03	11
GO:0007409	Axonogenesis	4.95	5.05E-03	6
GO:0032502	Developmental process	1.52	5.27E-03	29
GO:0048699	Generation of neurons	2.75	6.76E-03	10
**Molecular Function**				
GO:0008083	Growth factor activity	4.61	0.007009	6
GO:0005509	Calcium ion binding	1.85	0.074216	10
GO:0016705	Oxidoreductase activity, acting on paired donors, with incorporation or reduction of molecular oxygen	3.84	0.077651	4
GO:0004871	Signal transducer activity	1.61	0.099857	12
GO:0060089	Molecular transducer activity	1.61	0.099857	12
**Cellular Component**				
GO:0005576	Extracellular region	2.03	3.13E-04	24

The GO term and name are given, along with the total number of genes correlating with age in the category and fold enrichment (FE) of the GO term. For each GO category, the enriched categories or the top 10 is presented.

a Fold Enrichment of the GO term in the top list of genes (p<0.01; as shown in Table 2).

b Number of genes.

To further verify our findings, quantitative polymerase chain reaction (qPCR) was used to validate the gene expression of 12 genes in a selection of 20 donors from the total donor bank. These 12 genes (Table 4) were selected based on an earlier gene list, which was not yet filtered on standard deviation, nor corrected for sex or aspiration site. We used 10 male and 10 female donors, with 5 young and 5 old donors for each sex that were selected randomly.

**Table 4:** Gene expression validated with qPCR in 20 donors.

Official symbol	Position (ANOVA Table 2)	qPCR-validation P value	qPCR-validation R**
AUTS2	-	0.194	−0.303
* COL13A1	38	0.006	0.595
* COLEC12	7	0.044	−0.454
CCNG2	-	0.214	−0.291
DLK1	-	0.219	0.288
# FGFR2	3, 6	0.055	−0.435
* FST	2, 10	0.006	0.593
HOXB7	(181)	0.942	0.017
* JAG1	29, 37	0.006	0.593
JAG2	-	0.507	0.158
# SLIT3	1	0.088	−0.392
* ZNF395	-	0.004	−0.617

* represents p<0.05.

** R is the Pearson correlation value with donor age.

# validated on 61 donors (p<0.05) (Figure 3).

**Figure 3:**
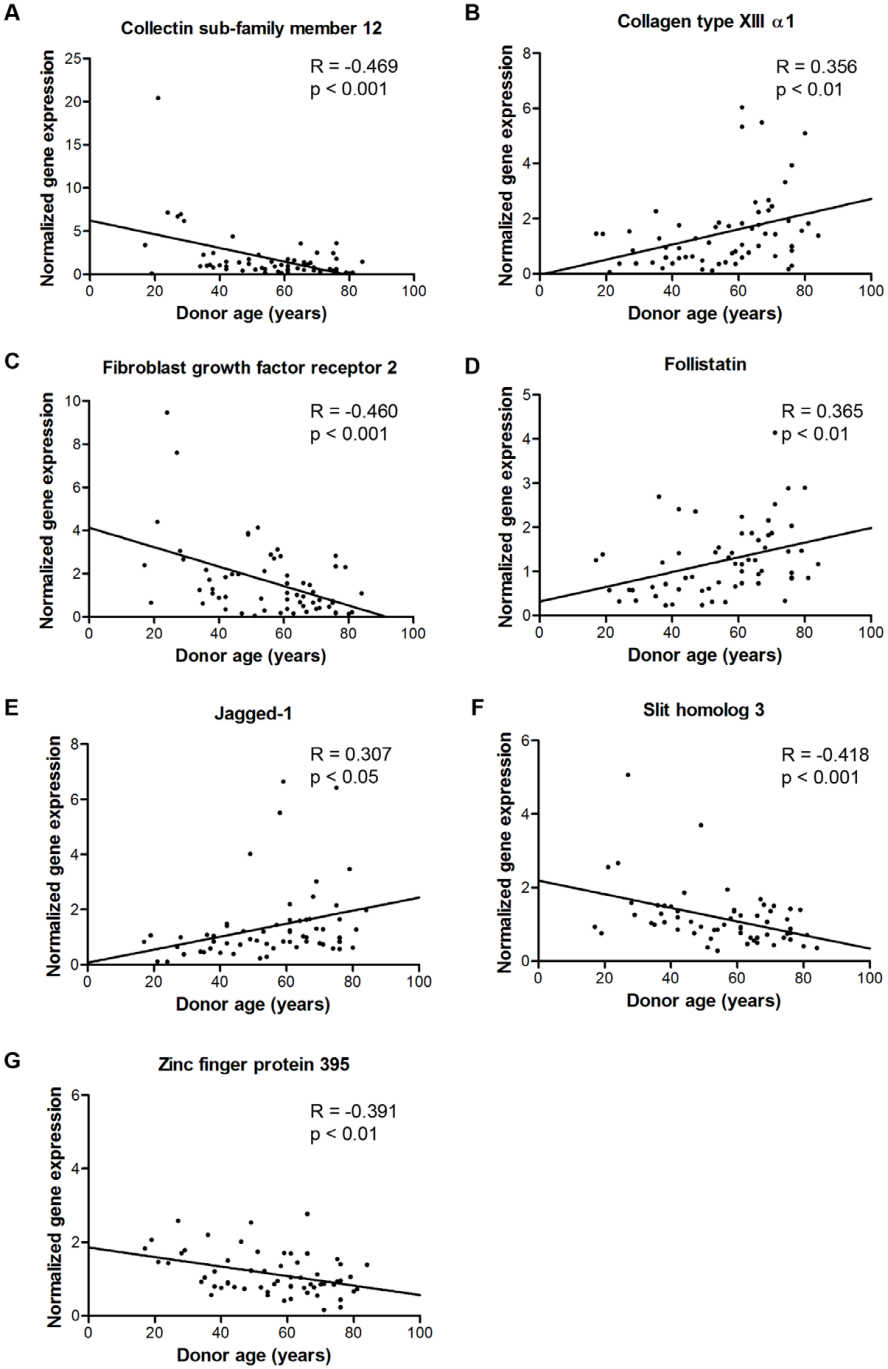
qPCR validation on the whole donor population. Correlation curves were produced for the expression of A) *COLEC12*, B) *COL13A1*, C) *FGFR2*, D) *FST*, E) *JAG1*, F) *SLIT3* and G) *ZNF395* in the entire donor population and significant correlations could be confirmed.

Of the 12 tested genes, 6 appear in the current top list (Table 2). The qPCR validation showed that 5 of those 6 genes were significant, and 2 nearly significant. A further extension of the qPCR testing to the full donor bank (61 donors) did show these latter 2 genes (Fibroblast growth factor receptor 2, *FGFR2* and Slit homolog 3, *SLIT3*) also to be significant (Figure 3). Of the 6 of 12 genes that are not in the current top list, 5 were removed by the standard deviation filtering. Of these genes, only 1 (*ZNF 395*) proved to be significant (note that the standard deviation filtering was decided on completely independent of the qPCR results). Indeed, the qPCR results did show that ZNF395 had a nearly flat correlation curve, especially when compared to collectin sub-family member 12 (*COLEC12*), which showed a 5-fold decrease in expression with age. The one remaining gene of the 6 genes not in Table 2, Homeobox B7 (*HOXB7*), was also not validated in the qPCR results. This gene was shown to actually correlate to the aspiration site (p-value 0,0002), and was removed from the top list by correcting it for aspiration location and sex. Homeobox B7 (*HOXB7*) was initially on the correlation list, but did not correlate with donor age based on the qPCR results. Based on the corrected correlation analysis, the expression of *HOXB7* was observed to be correlating to aspiration site and after correcting the ANOVA for aspiration site and sex, this gene was located at position 181 in the correlation list.

This correction was performed using multiple regression analysis, as depicted in Figure 1. For this, the following equation (Eq. 1) was used as described previously [19]:
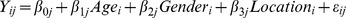



Although slightly different trends were observed in hMSCs obtained from male and female, gene expression of *COLEC12*, Collagen type XIII α1 (*COL13A1*), *FGFR2*, Follistatin (*FST*), *SLIT3* and *ZNF395*, solely, correlated with donor age (p<0.05). For these genes, correlation with age also gave p-values<0.05 when tested separately on hMSCs obtained from the acetabulum, as well as when tested on hMSCS obtained from the iliac crest. The expression of jagged-1 (*JAG1*) on the other hand, was significantly higher in hMSCs obtained from the acetabulum compared to hMSCs from the iliac crest. Concurrently, we only saw a significant correlation with age in the iliac crest (p-value 0.007), and not in the acetabulum (p-value 0.208). Multiple regression analysis was used to combine these seven genes in a general model for donor age according to the following equation (Eq. 2):
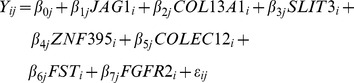
An R was reached of 0.673. When a small data set is used, the R^2^ has to be corrected, resulting in an adjusted R^2^. In this case the adjusted R^2^ is 0.381, in other words, 38.1% of the variance of donor age is explained by the combination of these seven genetic markers.

### Correlation of gene expression with *in vitro* senescence

**Figure 4:**
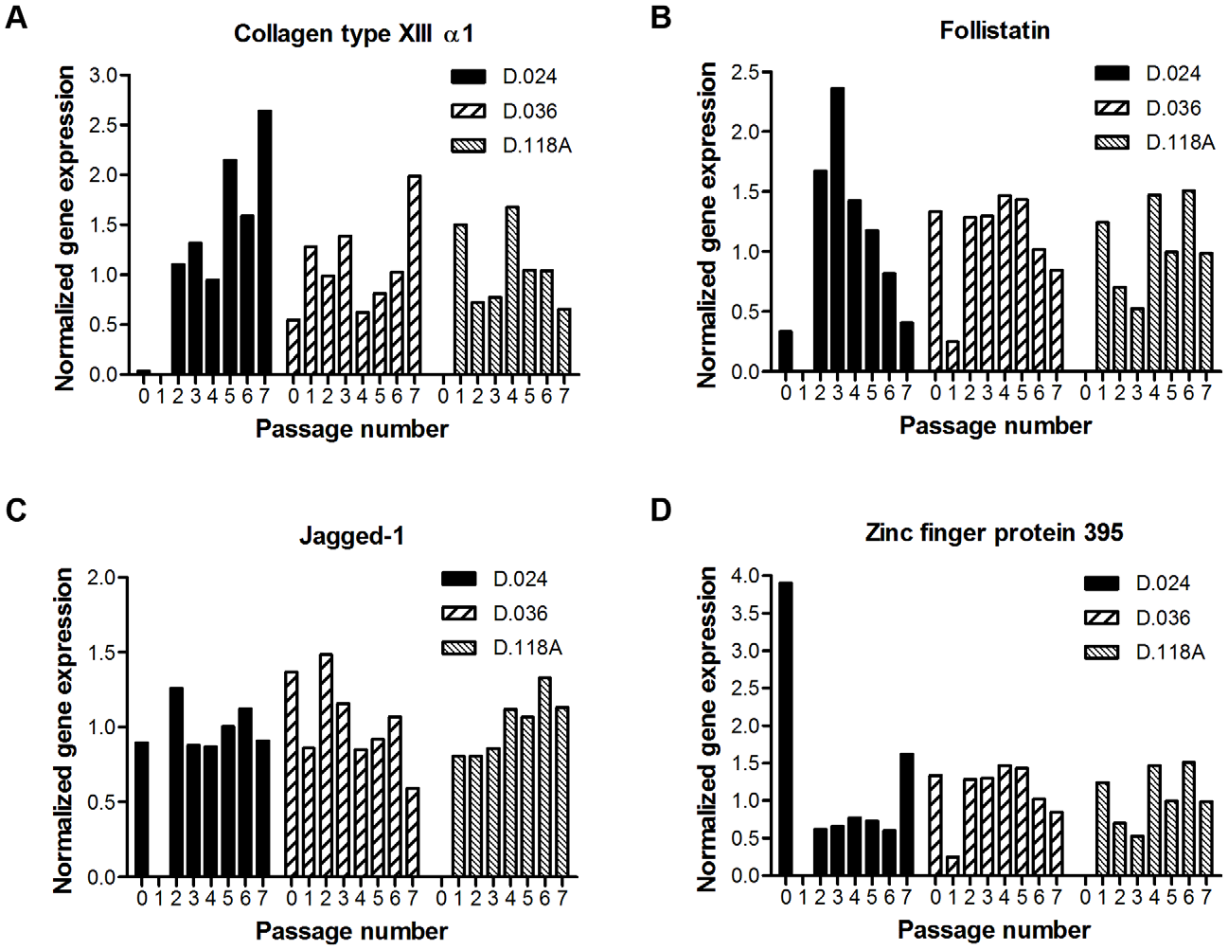
Gene-expression correlated to *in vitro* aging. The trend observed in relation to *in vivo* aging could not be confirmed in hMSCs aged *in vitro*. Gene-expression was determined up to passage 7 in three donors (D.024: female, 59 years, acetabulum; D.036: female, 38 years, iliac crest; D.118A: male, 60 years, acetabulum), however a general trend could not be observed. For some genes the expression pattern was random, A) *COL13A1* and D) *ZNF395*, and for other genes there seemed to be a trend in one of the donors, but this could not be confirmed in the other two, B) *FST* and C) *JAG1*.

An important question remaining is whether *in vitro* senescence of hMSCs reflects *in vivo* aging. It has been shown that long term *in vitro* expansion of hMSCs leads to senescence and that this process was associated with the accumulation of DNA damage [15], which is also associated to premature aging *in vivo* [16]. Since it is fundamental for the clinical application of hMSCs to have cells with high differentiation potential and with the least signs of aging, we investigated whether the genetic markers we discovered for *in vivo* aging, simultaneously were relevant for *in vitro* aging. Therefore, we performed qPCR on RNA isolated from hMSCs that have been serially expanded *in vitro* (3 donors, passage 0–7). Unfortunately, for the four genes we examined (with correlation p value<0.01 in the qPCR validation), consistent trends could not be determined (Figure 4). For *FST*, for example, its expression in D.024 shows a clear negative relation to passage number, however, this could not be confirmed in the other two donors.

### Correlation of gene expression and age in rat MSCs

**Figure 5:**
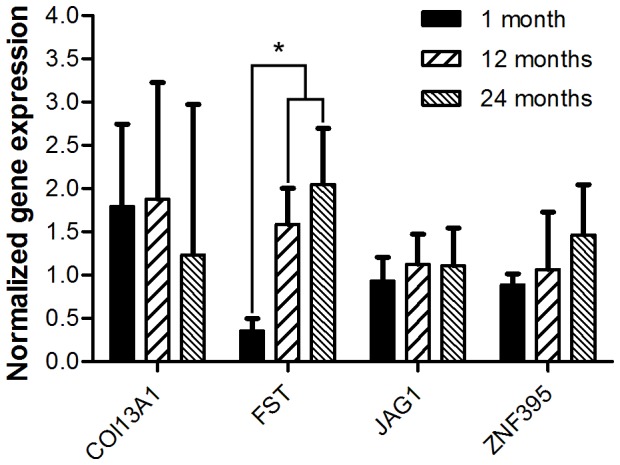
Validation of gene-expression in rat MSCs. To assess if age-related gene-expression was similar inter-species, MSCs were isolated from 1, 12 and 24 month old rats. The correlation between the expression of follistatin and age could be verified in rat MSCs, suggesting this is possibly a marker for several species. For the other three genes no correlation was found.

To validate if the genes we found are correlated to the mechanisms of aging in different species, we investigated their expression also in rat bone marrow stromal cells. To determine if these genetic markers could be used as markers of aging inter-species, we isolated MSCs from femora of young (1 month), adult (12 months) and old (24 months) Wistar rats. We have performed qPCR on four genes that were confirmed by qPCR to be significantly correlated with age (p<0.01). The age-related expression of *COL13A1*, *JAG1* and *ZNF395* could not be verified in rat MSCs. However, the expression of *FST* was significantly higher in MSCs from young rats than in old rats (Figure 5), consistent with our finding in human MSCs.

## Discussion

Aging is characterized by a series of progressive changes often associated with increased loss of tissue function and leading to increased risk of mortality. Many age-related changes are already described such as hair loss, decreased respiratory and kidney function, and decreased tissue regeneration which leads, for example, to an increased wrinkling of the skin. A single overall mechanism that gradually reduces functional capacity has not yet been identified even though the process of aging is likely to be genetically controlled by a small set of genetic mechanisms [21]. But if it is true that aging has a strong genetic component, it is also true that individual aging is subject to external influences as well. Stem cells are thought to be in the base of tissue regeneration by giving rise to progenitor cells that can differentiate and replace damaged cells and therefore delaying the onset of aging.

In this study, the first step taken in the search for a marker for aging was to evaluate whether chronological age had an effect on stem cells differentiation potential and proliferation capacity. For this, several biological parameters were then analyzed (Table 1) and correlated to age (e.g. population doublings per day in different passages, bone formation, ALP expression, adipogenesis, etc.). Most of these parameters did not correlate with age, except for the expression of bone-specific ALP (basic medium, P<0.05). This was mainly due to high expression of ALP in a number of young donors and therefore, a significant general trend was obtained. However, the osteogenic differentiation capacity determined by dexamethasone-induced ALP expression, mineralization and *in vivo* bone formation did not correlate in any way.

Since the correlation obtained was only mildly significant, in order to find a better marker for aging, we have generated a high-resolution transcriptional profile of aging in human mesenchymal stromal cells using a large sample size of 61 donors, with ages ranging from 17–84 years. Previously, the effect of *in vitro* senescence was investigated on different hMSC populations derived from one young donor (15 years old) [22]. In this study, the authors identified 338 genes related to *in vitro* senescence. Interestingly, 6 genes overlapped with our top 70 list correlating to *in vivo* aging: *CDH13, ZNF 365, ANKRD1* (ankyrin repeat domain 1 (cardiac muscle), *COLEC12*, *INSIG1* (insulin induced gene 1) and *CDH6* (cadherin 6, type 2, K-cadherin (fetal kidney)). In contrast, the observed down regulation of *KRT18 in vitro* could not be confirmed, this gene was positively correlated with age in our dataset. Furthermore, another gene expression profiling study on hMSCs has been previously performed, however, on a limited donor population (young, median aged and elderly donors, n = 4 per group) [23]. Their analysis revealed 184 genes correlating with age, of which, only *S100A4* is present in our list. Although *HOXB7* and *SHOX2* were present in our initial list as well, similar to their results, the expression significantly correlated with the aspiration site of bone marrow in our study. Our work provides, however, a significantly larger sample size which enables a higher statistical resolution and further evaluates the relation between donor age and MSC differentiation capacity, compares the expression of the markers obtained by the gene expression profiles with *in vitro* replicative senescence and assesses whether the same markers can predict the chronological age also in animal models (Wistar rats).

Other studies have focused on the characterization of tissue-specific gene expression profiles such as: human brain [17], human muscle [18] or human kidney [19]. Similar studies have also been done in a large Epstein-barr virus-immortalized lymphoblastoid cell line bank (46 three-generation families) [24] and in multiple mouse strains [25].

The main advantage of our work is that a whole genome-wide gene expression profiling was performed in a large sample size of untransformed hMSCs (61 donors) and since it was performed on human MSCs, it allowed us to obtain general markers for aging rather than tissue-specific ones, which are more prone to present higher donor variation due to external factors since in our study the co-founder effects can be more easily evaluated. While some patterns of aging are similar between human tissues, much of it is tissue specific [18].

Furthermore, examination of the gene ontology (GO) terms on the top 70 gene list revealed an overrepresentation of GO terms related to neuronal functioning. Furthermore, more general terms were enriched like growth factor activity and located in the extracellular region. A number of genes could be linked to hMSC function or aging. *FGFR2* is targeted by bFGF and positively regulates osteogenic differentiation [26] and as expected, *FGFR2* expression declines with donor age. Ankyrin 3 (*ANK3*) is overexpressed in cells with the progeroid syndrome Hutchinson-Gilford [27], which is supported by the increase of gene expression with donor age in our research. The expression of *JAG1*, part of the Notch signaling pathway and essential for the regeneration of muscle, is impaired in aged human muscle tissue [28]. In addition, *JAG1* enhances the potential of MSCs to differentiate into cardiomyocytes [29]. We expected *JAG1* expression to decrease with donor age, however, our results prove the contrary, suggesting a different function for Notch signaling in MSCs compared to muscle. Notch receptors are however, known to regulate cell fate determination, stem cell self-renewal, proliferation and apoptosis and *JAG1* was previously associated with a poor prognosis for breast cancer patients [30]. Therefore, it could be associated with the increased chance of cancer occurrence in elderly patients.

The aspiration site correlates significantly with donor age (correlation value = 0.4103 and p-value = 0.0021). This variable was considered, together with donor sex, as confounding factor in the ANOVA model. The genes listed in Table 2, with a correlation p value<0.01, were tested on their correlation between aspiration site and donor age. Eleven genes from this list were significantly affected by the aspiration site, which could not be proven for the other genes in the list. Nevertheless, the correlation between the listed genes and donor age could in many cases be observed for both aspiration sites individually (data not presented). Furthermore, the dataset was filtered based on standard deviation over all donors in order to limit the data to probe sets with larger effect sizes. Some genes that correlated with donor age in an initial gene list, were filtered out due to their low effect size. For example, *ZNF395*, included in the validation qPCR, showed a low but significant correlation (Figure 3G) while this gene was filtered out by the adjusted analysis method. Some genes occur more often in the top 70 probe-set list, making them more powerful indicators for donor age. Moreover, false positive rates were calculated, showing that the number of false positive probes that can be expected is relatively limited. qPCR validation confirmed our microarray analysis as a reliable method for gene expression studies. Moreover, we have shown that *FST* is also differently expressed during age in rat MSCs, indicating it as a possible inter-species marker for age. FST has first been identified as an indirect regulator of follicle-stimulating hormone secretion by binding irreversibly to activin and thus inactivating it. Klein *et al.* have shown that FST concentrations in follicular fluids from older female subjects (40–45 year) were significantly higher compared to younger female subjects (20–25 year) [31]. Furthermore, FST is known to bind to myostatin. Increasing FST levels are also identified in muscle biopsies from older male subjects [32]; this effect could be counteracted by eccentric exercise [33]. This might be due to increases in myostatin levels associated with muscle fiber atrophy, although this could not be confirmed in our assay. Additionally, FST has been identified as both a stimulator [34] and inhibitor of bone mineralization [35]. A recent study in mice demonstrated potential consequences of the increased levels of FST with age; overexpression of FST led to reduced mechanical properties of bone and therefore increased the susceptibility to fractures [36]. Altogether, FST has the potential to regulate aging in multiple tissue types.

Despite it was reported that several MSC age-related gene expression changes (especially genes involved in genomic integrity and regulation of transcription that were age-repressed) were also differentially expressed during *in vitro* senescence [22], [23], we haven't seen the same in our study. Our aging markers do not apply to *in vitro* senescence since no correlation was found amongst the three donors tested. Similar results were obtained previously where it has been shown that the expression signature of *in vitro* senescence resembled mouse but not human aging [37].

Our results showed a large donor variation in gene expression during *in vitro* replicative senescence and therefore, suggested that data obtained *in vitro* might not be entirely correlated with what happens *in vivo*. Although both intrinsic and extrinsic factors might contribute for *in vitro* senescence, it is likely that the adverse culture conditions influence more the *in vitro* senescence process, presumably making hMSCs more exposed to DNA damage than they would face *in vivo*.

In this study we were able to identify genes that significantly correlated with donor age, suggesting that stem cells are also influenced by the aging process. Interestingly, we have observed a remarkably small effect of donor age on the biological characteristics tested (Table 1), which shows that MSCs from the bone marrow seem to maintain their quality with aging. This is in agreement with the hypothesized fact that stem cells themselves seem to be protected from aging due to their mostly quiescence status, however, in contradiction with the studies on aging hematopoietic stem cells (HSCs) where it was demonstrated that although the repopulation ability of HSCs from young versus old follow the same time frame, some months after transplantation the contributions from the old HSCs drop considerably, which suggests that aging HSCs lose their repopulating capacity [38]. If there are some disparities between HSC and MSC aging effects on their quality and performance, little disagreement exists about the fact that regenerative potential and body repair systems deteriorate with age.

Another interesting aspect was the fact that there is a clear large inter-donor variability present on MSCs from the same age, even though aging itself has no clear effect on performance. Unfortunately we do not have the disease history of the patients, so we were not able to analyze if the potential orthopaedic disease history might be of influence in the data analysis.

Our top list with genes correlating to *in vivo* donor aging, may provide a window of opportunity to acquire more insight into the molecular mechanisms behind human aging.

In conclusion, we have identified a set of genes which expression in human MSCs correlates to donor age but not to *in vitro* senescence. The presence in the list of genes known to be involved in aging, demonstrates the biological relevance of the genes. The new genes correlated to age unveiled in this manuscript may lead the way to a better insight into the process of human MSC aging.

## Materials and Methods

### Isolation and culture of human mesenchymal stromal cells

Bone marrow aspirates (5–20 ml) were obtained from donors with written informed consent - for under aged donors legal guardians were involved - and were approved by the medical ethical committee of the University medical Centre Utrecht, and hMSCs were isolated and proliferated as described previously [39]. Briefly, aspirates were resuspended using a 20-gauge needle, plated at a density of 500,000 nucleated cells/cm^2^ and cultured in hMSC proliferation medium containing α-minimal essential medium (α-MEM; Gibco), 10% fetal bovine serum (FBS; Biowhittaker, Australia), 0.2 mM ascorbic acid (Sigma), 2 mM L-glutamine (Gibco), 100 U/mL penicillin with 100 µg/mL streptomycin (Gibco) and 1 ng/mL basic fibroblast growth factor (bFGF; Instruchemie, Delfzijl, The Netherlands). The serum batch was selected on proliferation rate and osteogenic differentiation potential and used for all experiments in this paper. Cells were grown at 37°C in a humid atmosphere with 5% CO_2_. Medium was first changed after 5 days to remove non-adherent cells and was further refreshed twice a week. Cells were used for further subculturing or cryopreservation upon reaching near confluence. hMSC basic/control medium (BM) was composed of hMSC proliferation medium without bFGF, hMSC osteogenic medium was composed of hMSC basic medium supplemented with 10^−8^ M dexamethasone (dex, Sigma). Basic medium was used as a control. ALP expression was measured by flow cytometry as described previously [40].

### Isolation and culture of rat mesenchymal stromal cells

Rat MSCs were obtained as described previously [41]. Femora of 1, 12 and 24 months old male rats (Male Crl:WI(Han), Charles River, n = 3) were removed, thoroughly cleaned and submerged in PBS containing 250 µg/ml Fungizone (Invitrogen). After removal of the epiphyses, the bone marrow was flushed out with 10 ml of rat MSC proliferation medium containing α-MEM (Gibco), 15% FBS (Biowhittaker, Australia), 0.2 mM ascorbic acid (Sigma), 2 mM L-glutamine (Gibco), 100 U/mL penicillin with 100 µg/mL streptomycin (Gibco) and 1 ng/mL bFGF (Instruchemie, Delfzijl, The Netherlands) and cultured in T75 flasks. Cells were grown at 37°C in a humid atmosphere with 5% CO_2_. Medium was first changed after 3 days to remove non-adherent cells and was further refreshed three times a week. Animals were housed at the Central Laboratory for Animal Institute (Utrecht University, Utrecht, The Netherlands), and experiments were approved by the local animal care and use committee.

### Microarray analysis

To analyze the gene expression profile of hMSCs, cells were seeded at 1000 cells/cm2 and upon reaching near confluence RNA was isolated using an RNeasy mini kit (Qiagen) and DNase treated on column with 10 U RNase free DNase I (Gibco) at 37°C for 30 minutes. DNase was inactivated at 72°C for 15 minutes. The quality and quantity of RNA was analyzed by gel electrophoresis and spectrophotometrically. The RNA was hybridized to the Human Genome U133A 2.0 Array (Affymetrix) and scanned with a GeneChip G3000 scanner (Affymetrix). The microarray experiments were performed in three batches. Although this was done at the same microarray facility using arrays from the same production batch, there were still noticeable batch effects. To normalize the measurements, we used a normalization method which removes hybridization, amplification and array location effects [42]. Afterwards, probe sets which did not show significant differences in expression between arrays (std<0.4) were removed. The remaining 1653 probe sets (out of 22,277 probe sets) were used for further analysis. To determine the most significant probe sets with respect to age, we determined a p-value for each gene by permuting F-test scores. In total, 10^5^ permutations were performed for each gene. To adjust for multiple testing, we calculated a false discovery rate. Microarray data can be retrieved from the Gene Expression Omnibus (GEO) at the National Center for Biotechnology Information (NCBI) under the accession number GSE39540. Enriched biological terms were identified by Gene ontology analyses on the genes correlating with age (p<0.01; as presented in Table 2) using the Database for Annotation, Visualization and Integrated Discovery (DAVID) [43].

### Quantitative polymerase chain reaction (qPCR)

To investigate gene expression, hMSCs were seeded at 1000 cells/cm^2^ and freshly isolated rat MSCs were used, upon reaching near confluence RNA was isolated using an RNeasy mini kit (Qiagen) and DNase treated on column with 10 U RNase free DNase I (Gibco) at 37°C for 30 minutes. DNase was inactivated at 72°C for 15 minutes. The quality and quantity of RNA was analyzed using gel electrophoresis and spectrophotometrically. The iScript cDNA synthesis kit (Bio-rad) was used according to the manufacturer's protocol to synthesize first strand complementary DNA (cDNA) from 1 µg RNA. qPCR was carried out on a iQ™5 Real-Time PCR Detection System (Bio-Rad) using 1 µL of 1×, 10× or 100× diluted cDNA, 500 nM forward primers, 500 nM reverse primers, 2× iQ SYBR Green Supermix (Bio-rad). For the gene expression analysis of *AUTS2*, *DLK1* and *JAG2*, the forward and reverse primers were substituted for 1 µL gene-specific RT^2^ qPCR Primers (SABiosciences). The remaining primer sequences (Sigma) are depicted in Table 5, as a reference gene GAPDH was used for hMSCs and β-actin for rat MSCs. Primer efficiencies were determined and gene expression was calculated and normalized to the average gene expression according to the method of Pfaffl et al. [44].

**Table 5:** qPCR primer sequences.

Gene name	Forward primer (5′-3′)	Reverse primer (5′-3′)
hGAPDH	cgctctctgctcctcctgtt	ccatggtgtctgagcgatgt
hCOLEC12	actcagagcgtgaaaatgaatgg	cccagcataaatcaacccagc
hCOl13A1	ttggatccggtcaaccaggcactagaggtttcc	ttgaattcttggatgctggcctggctctgttcg
hCCNG2	ccaacttctcgggttgttgaacgtctacc	ctaatccggatcacatcatgagtg
hFGFR2	ggctgccctacctcaaaggttc	agtctggggaagctgtaatctc
hFST	aggaggacgtgaatgacaaca	ccaaccttgaaatcccataaa
hHOXB7	agagtaacttccggatcta	tctgcttcagccctgtctt
hJAG1	aggccgttgctgacttagaa	gcagaagtgggagctcaaag
hSLIT3	ccgcctaactacacaggtgagctat	cgctgtagccagggacacact
hZNF395	agagtctggggctgtgtgtt	atggtccttttgctttgcac
rβ-actin	ttcaacaccccagccatgt	tgtggtacgaccagaggcatac
rCOL13A1	accgggggcctctgggatt	ggcagggggcgtctagtcca
rFST	cggctgagcacctcgtggac	tcggcactttttcccggggc
rJAG1	ggtgtggcccgagaccttgc	gctggaggctggaggaccga
rZNF395	ctgtgtgccaggagcagccc	ctgctccaccaggcccttgc

### Statistics

Regression analysis and Pearson correlations were used to analyze correlations between donor age and biological parameters and gene expression. Multiple regressions were used to exclude sex and location of aspiration as confounding factors (Eq. 1) and to analyze if the combinations of multiple genes gave a more accurate description of the age of the donor (Eq. 2).
